# The Properties and Applicability of Bioprinting in the Field of Maxillofacial Surgery

**DOI:** 10.3390/bioengineering12030251

**Published:** 2025-03-01

**Authors:** Luca Michelutti, Alessandro Tel, Massimo Robiony, Shankeeth Vinayahalingam, Edoardo Agosti, Tamara Ius, Caterina Gagliano, Marco Zeppieri

**Affiliations:** 1 Clinic of Maxillofacial Surgery, Head-Neck and NeuroScience Department, University Hospital of Udine, p.le S. Maria della Misericordia 15, 33100 Udine, Italy; micheluttiluca.uniud@gmail.com (L.M.); alessandro.tel@icloud.com (A.T.);; 2 Radboud University Medical Center, 6525 GA Nijmegen, The Netherlands; 3 Division of Neurosurgery, Department of Medical and Surgical Specialties, Radiological Sciences and Public Health, University of Brescia, Piazza Spedali Civili 1, 25123 Brescia, Italy; 4 Academic Neurosurgery, Department of Neurosciences, University of Padova, 35121 Padova, Italy; 5 Department of Medicine and Surgery, University of Enna “Kore”, Piazza dell’Università, 94100 Enna, Italy; 6 Mediterranean Foundation “G.B. Morgagni”, 95125 Catania, Italy; 7 Department of Ophthalmology, University Hospital of Udine, 33100 Udine, Italy; 8 Department of Medicine, Surgery and Health Sciences, University of Trieste, 34100 Trieste, Italy

**Keywords:** bioprinting, regenerative medicine, personalized treatment, maxillofacial surgery

## Abstract

Perhaps the most innovative branch of medicine is represented by regenerative medicine. It deals with regenerating or replacing tissues damaged by disease or aging. The innovative frontier of this branch is represented by bioprinting. This technology aims to reconstruct tissues, organs, and anatomical structures, such as those in the head and neck region. This would mean revolutionizing therapeutic and surgical approaches in the management of multiple conditions in which a conspicuous amount of tissue is lost. The application of bioprinting for the reconstruction of anatomical areas removed due to the presence of malignancy would represent a revolutionary new step in personalized and precision medicine. This review aims to investigate recent advances in the use of biomaterials for the reconstruction of anatomical structures of the head–neck region, particularly those of the oral cavity. The characteristics and properties of each biomaterial currently available will be presented, as well as their potential applicability in the reconstruction of areas affected by neoplasia damaged after surgery. In addition, this study aims to examine the current limitations and challenges and to analyze the future prospects of this technology in maxillofacial surgery.

## 1. Introduction

### 1.1. Three-Dimensional Printing

Three-dimensional printing, an additive manufacturing method, is extensively utilized in numerous sectors, particularly in healthcare. After Chuck Hull filed his patent in the 1980s, the entry of this technology into the industrial sector led to an exponential growth of 3D printing by attracting more and more funding, research, and technological innovation [1]. With the entry and diffusion of 3D printing into the medical sector about a decade ago, the ability to print three-dimensional models constituted a great advantage, particularly in the surgical field. In fact, thanks to it, it is possible to make prostheses, customized devices, and implantable medical devices, but also to create 3D models that can help the surgeon to better and more accurately plan a surgical procedure, leading to a reduction in operating time and peri-operative complications. It is important to mention the enormous educational potential that printing three-dimensional models can have to teach young surgeons and future physicians through the simulation of surgical procedures by means of three-dimensional printed models, ensuring improved performance and skills [2,3,4,5]. This is precisely why 3D printing is finding wide application in different branches of medicine, such as in maxillofacial surgery, to help clinicians better deal with different pathologies, specifically in the management of trauma and oncological diseases. All of this is with a view toward an increasingly personalized and precision medicine [6,7,8,9].

The domain of reconstructive surgery has been transformed by patient-specific implants (PSIs) produced through 3D printing. These implants, customized to meet specific patient requirements, are produced utilizing imaging data from CT or MRI scans to generate a precise three-dimensional model of the problem site. Hydroxyapatite (HA) and bone cement (polymethylmethacrylate, PMMA) are often utilized materials for PSIs, recognized for their biocompatibility and osteoconductive characteristics. Hydroxyapatite, a calcium phosphate ceramic, closely mimics the mineral composition of bone, enhancing integration and cellular adhesion. Bone cement functions as a rapid-setting substance suitable for fixing bone deformities and securing prosthetic devices. Notwithstanding their benefits, difficulties persist, such as mechanical brittleness in HA and thermal impacts during PMMA polymerization. Addressing these constraints via sophisticated material engineering and hybrid methodologies is essential for broadening their therapeutic applications [10,11,12,13].

The applications of 3D printing in maxillofacial surgery are manifold, in orthognathic surgery, in bone reconstruction, in the treatment of TMJ disorders, and all through the realization of splints, 3D bone models, surgical guides, PSIs, and scaffolds [14]. Surgical guides are useful tools for performing osteotomies in orthognathic surgeries and for patient-specific fibula retrievals; PSIs are tools that aid in the reconstruction of zygomatic deformities, for the reconstruction of orbital fractures, and for reconstruction following maxillectomy; and splints prove essential for guiding orthognathic surgery to achieve a correct occlusion (Table 1) [14,15,16,17,18,19,20].

The field of 3D printing has matured to the point where it is widely used to produce PSIs (patient-specific implants) with customized mechanical properties and precise geometries based on patient-specific anatomical features. The fabrication of implants has been made possible by the development of biocompatible and bioactive materials, including PEEK (polyetheretherketone), PMMA (polymethylmethacrylate), and titanium alloys, and by the evolution of printing techniques, with increasingly efficient and widespread machinery. At the same time, bioprinting technologies are evolving to include multi-material printing, vascularization strategies, and bioactive factors. These advances point to a possible future in which bioprinting could generate fully functional composite tissues and organs for clinical applications, such as transplants or reconstructions of complex anatomical areas. However, substantial research and technological advances are needed to address current obstacles, especially because bioprinting, unlike 3D printing, aims not only to replace lost anatomical structures but also to regenerate them [21,22,23].

### 1.2. Bioprinting: The Fusion of 3D Printing and Regenerative Medicine

In recent years, research has increasingly focused its attention on bioprinting, that is, a technology that can provide the ability to print anatomical tissue structures using living cells, scaffolds, and molecules, capable of making a tissue that is histologically the same as human tissue. This stems from the need to make tissues to repair those damaged and degenerated by pathological and physiological processes such as aging. The possibility of creating tissues with autologous cells taken from the diseased patient to replace damaged organs would mean eliminating the risk of rejection that is present when transplantation is necessary [10,24,25].

Regenerative medicine, along with tissue engineering, i.e., the joining of bioactive materials with living cells, is a branch of medicine that aims to make these tissue substitutes, such as bone tissue, blood vessels, cartilage, and liver, to regenerate damaged human tissues and for research purposes. In fact, the fabrication of in vitro tissues makes it possible to study the pathogenesis of many diseases through cellular models, investigate the effects of gene expression of tumors, and study the effectiveness of targeted therapies against neoplastic diseases [26,27].

The fusion of 3D printing and tissue engineering thus opens a new frontier in regenerative medicine, namely bioprinting. Regenerative medicine, along with tissue engineering, is a branch of medicine dedicated to the development of tissue substitutes, including bone tissue, blood vessels, cartilage, and liver, with the goal of regenerating damaged human tissues and conducting research, particularly on the mechanisms of carcinogenesis of various cancers or the development of new therapeutic and pharmacological approaches. The fusion of tissue engineering and 3D printing has ushered in a new era of regenerative medicine and bioprinting. This multidisciplinary approach integrates the complexity of tissue engineering with the precision of 3D printing, thus offering new opportunities for personalized medicine [26,28,29].

**Figure 1:**
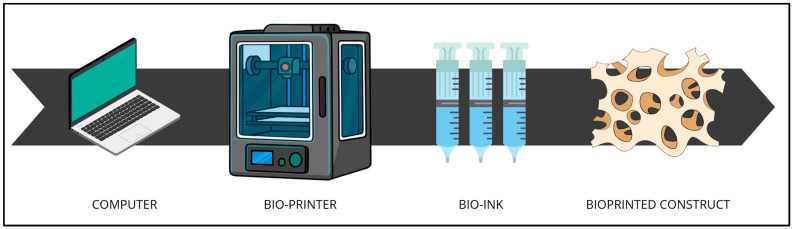
A schematic representation of the key elements needed for bioprinting. Considering the multiple steps and processes, one understands the need for a multidisciplinary team consisting of physicians, engineers, biologists, and computer scientists [30] (image created utilizing Canva^®^).

The bioprinting was described by Murphy and Atala as “layer by layer precise positioning of biological materials, biochemicals and living cells with spatial control of the placements of functional components to fabricate 3D structures” [25]. This technology requires multiple professionals precisely because of its complexity. A number of processes and processing steps characterize it. In fact, bioprinting requires bioinks, i.e., special materials that contain live cells and other biological components, a 3D printer capable of depositing the bioink layer by layer, a digital 3D model of the organ or tissue to be made, live cells that are mixed with the bioink, nutrients and growth factors that promote cell growth, and bioreactors, which are essential for creating a controlled environment aimed at cell growth and differentiation and for tissue development (Figure 1) [30].

It can already be understood that it is a multidisciplinary technology. That is, multiple skills are required in the fields of biology, chemistry, engineering, and medicine, and it requires the involvement of multiple professionals, such as physicians, engineers, computer scientists, and biologists.

### 1.3. Objective of This Study

Given the strong growth of bioprinting that has been emerging more and more in recent years, this review aims to provide some background on how this technology works and to present a general overview of what may be potential applications of bioprinting in the field of maxillofacial surgery, analyzing the main printing techniques, the most commonly used biomaterials, the most important current obstacles that limit its current applicability in clinical reality, and make possible proposals for future research.

The aim of this review is to deliver a thorough and up-to-date examination of the characteristics and uses of bioprinting in cranio-maxillofacial surgery. This review intends to provide a comprehensive examination of bioprinting applications in cranio-maxillofacial surgery, emphasizing recent breakthroughs, existing constraints, and prospective potential.

## 2. Bioinks

### 2.1. Components of Bioinks

Bioinks are the raw materials required for making 3D bioprinting, they are a formulations of biomaterials and living cells. They are deposited by special machines layer by layer and are the basis for the fabrication of complex biological structures such as tissues and organs. These inks consist of living cells and biomaterials, potentially hydrogels, liquid biomaterials consisting of polymers that can form a three-dimensional network with supporting functions. In addition, within these inks are biomolecules and growth factors that are essential for the growth and survival of the living cells they contain. They are, therefore, made up of different biomaterials in order to keep the cells united and encapsulated and retain the biomolecules. The bioinks are characterized by different properties including physical, rheological, mechanical, and biological ones [30,31].

**Figure 2:**
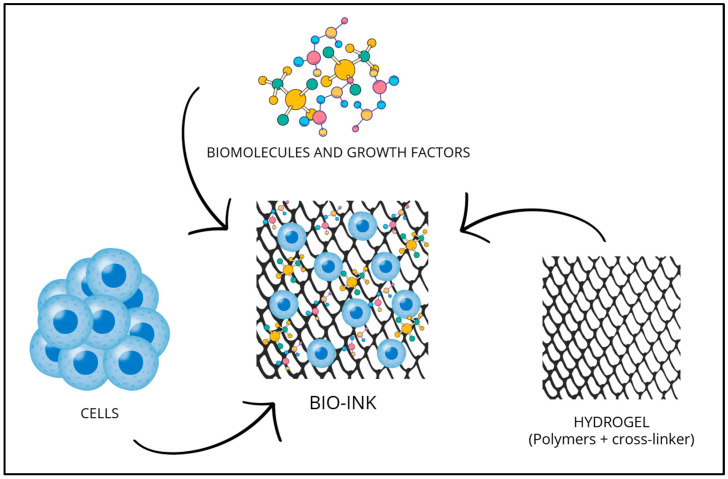
The main components of bioinks. They consist of living cells and a three-dimensional network called a hydrogel [32,33,34] (image created utilizing Canva^®^).

The first generation of 3D bioprinters used biomaterials to make scaffolds, structures with support purposes, on which living cells were later seeded to make tissue. Unfortunately, this mode of printing results in the uneven seeding of cells. To achieve a homogeneous distribution of cells, biomaterials containing living cells directly within them were made, i.e., bioinks (Figure 2) [32,33,34].

### 2.2. Biomaterials and Hydrogels

Hydrogels are materials consisting of polymer chains that can form a three-dimensional network capable of trapping copious amounts of water. Polymeric hydrogels are a class of biomaterials with the purpose of recreating the tissue microenvironment to control cell growth. In fact, they can encapsulate living cells and impart support to them by sending chemical and physical signals that can determine proper tissue formation. Together with living cells and biomolecules, these materials constitute bioinks and have the function of imparting mechanical and biological properties to printed tissues [35,36,37].

The polymer chains are organic biomaterials with high water contents for the hydration of the printed construct and for supporting cell functions and tissue regeneration. There are different types of polymers—either natural or synthetic. The first ones are able to support cell functions, while the latter are biologically inert but mechanically strong [38]. For micro-extrusion bioprinting, the most widely used printing technique, the naturally derived bioinks include alginate, agarose, type I collagen, gelatin, hyaluronic acid, fibrin and decellularized extracellular matrix, and the synthetic bioinks include poly-ethylene glycol (PEG), poly-ε-caprolactone (PCL) and poly-ethylene oxide (PEO) [39].

#### 2.2.1. Natural Polymers

The bioink could have mechanical and biological properties, and in addition to maintaining cell viability, it must be printable [40,41]. To meet all the essential properties that a bioink must have, these are often composed of multiple components, and unlike those consisting of only one component, they prove superior in terms of mechanical and functional characteristics [40,42]. The main characteristics of the major natural polymers are summarized in Table 2.

**Alginate** is a soluble polysaccharide and us composed of beta-D-mannuronic acid and alpha-L-guluronic acid. It is a biomaterial commonly used in extrusion printing because of its easy printability, water-absorbing ability, and because of its low cost. It has also demonstrated great compatibility with numerous tissues including bone, muscle, skin, nerves, vessels, and cartilage. Unfortunately, alginate has a poor cell-adhesion capacity due to the lack of adhesion molecules [43,44,45].

**Agarose** is a natural polysaccharide controlled by temperature. This type of biomaterial has limited cell-support functions and is often used as a “sacrifice biomaterial” in the realization of vascularized scaffolds. In fact, by taking advantage of temperature, agarose fibers are eliminated and removed. In fact, to impart more cell-support capacity, it is added with other materials such as alginate or collagen [46,47,48].

**Collagen** consists of numerous polypeptide chains. It is a material capable of influencing cell adhesion and for this reason often used to make tissue scaffolds. Type I collagen is the most widely used and is a material that can be controlled through pH and temperature modification. Collagen is used in various tissues but has the limitations of having poor mechanical properties. Again, combination with other materials, such as alginate or gelatin, can confer greater mechanical strength [49,50,51].

**Gelatin** has low immunogenicity, biodegradability, and good tissue compatibility. It can also be controlled by temperature. To use this type of biomaterial, other chemicals, such as methacrylate or glutaraldehyde, are used to improve stability, making a modified gelatin that can be manipulated through the use of UV light in order to polymerize it and encapsulate cells within it. The gelatin-meth-acryloyl (GelMA) can be used to make various tissues including bone, skin, cartilage, and vascular structures [52,53,54].

**Chitosan** is a polysaccharide derived from chitin. It is a material with antibacterial, biodegradable, and nontoxic properties, plus it is relatively inexpensive. It is a material that is soluble in acidic solutions and therefore not suitable for cell survival. To overcome this problem, it can be chemically modified by making it soluble in water with a neutral pH. Unfortunately, this biomaterial has several limitations such as poor mechanical properties for bioprinting, which can be overcome by the addition of additional hydrogels (based on gelatin, collagen, or alginate) that increase the rate of polymerization and mechanical strength [55,56,57,58].

**Hyaluronic acid** is one of the main components found in the extracellular matrix. It is a glycosaminoglycan that can attract a lot of water and is particularly viscous, such that it is used to adjust the viscosity of other biomaterials. It exhibits good adaptability for the cells contained in the bioink due to its adjustable physical properties. A particular type of hyaluronic acid is gelled hyaluronic acid, which unfortunately has poor mechanical properties. To overcome this limitation, it can also be combined with methacrylate, which can be polymerized by UV light [59,60,61].

**Fibrin** is a protein involved in both extracellular matrix formation and coagulation. To obtain fibrin, the same process that occurs naturally during coagulation is carried out artificially by adding thrombin and factor XIII to fibrinogen. Fibrin scaffolds allow for good cell-adhesion capacity; however, their use is limited by poor mechanical properties and rapid degradation. However, the latter limitation can be addressed by modifying the biomaterial through different concentrations of fibrinogen and calcium ions, through cell density or temperature [62,63,64,65].

**A decellularized extracellular matrix** is a biomaterial that is obtained through the removal of cellular components from native tissues by the process of decellularization. Once the cellular components are removed, biomolecules, growth factors, and proteins normally present in the extracellular matrix (fibrin, collagen, glycosaminoglycans) remain. By analyzing the components of this matrix, one can understand the good functional support capacity of the cells. Unfortunately, the main limitation of this biomaterial is mechanical weakness, although this can be remedied by combination with other polymers that confer greater stability and mechanical strength. In addition, this type of matrix can be applied to make skin, liver tissue, heart tissue, bone, cornea, and tendons [66,67,68,69,70].

#### 2.2.2. Synthetic Polymers

These types of biomaterials are not suitable for the direct incorporation of viable cells, unlike natural polymers, both because of their physical and chemical characteristics and because of the conditions required for their printing, which are prohibitive to viable cells (excessively high temperatures, use of solvents, etc.). Nevertheless, they prove very useful because of their high mechanical properties. Generally, these biomaterials are used to create scaffolds into which, at a later stage, hydrogels containing viable cells are bioprinted. Doing so gives the molded construct greater mechanical strength, a feature that is not always present in uniquely natural-based biomaterials [71]. The main characteristics of the major synthetic polymers are summarized in Table 3.

**Poly-ethylene glycol** (PEG) and **poly-ethylene oxide** (PEO) are polyethylene-based polymers and are the most commonly used synthetic hydrogels in bioprinting. They exhibit many useful characteristics, for example, the ability to bind water molecules, low immunogenicity, and biocompatibility. They also enable, due to their permeability, exchanges of nutrients and waste metabolites. Unfortunately, because of their synthetic nature, they have poor cell adhesion, and to overcome this problem they can be combined with peptides that can provide this essential feature. These hydrogels are used for the creation of cell scaffolds [72,73,74].

**Poly-ε-caprolactone** (PCL) is a thermoplastic, durable, and biodegradable polyester. Given that high temperatures are required to print this material, cell seeding occurs later to support cell viability. This is a material that is often combined with other natural polymers to impart greater mechanical strength, and it is mainly used for making bone tissue [75,76,77].

## 3. Printing Techniques

There are many printing technologies used in bioprinting to make scaffolds, such as stereolithography, selective laser sintering, fused deposition modeling, inkjet printing, and micro-extrusion. Stereolithography is one of the printing methods that involves the deposition of thin layers of material that are cured by UV light. This method, introduced by Charles W. Hull in 1986 (PubChem Patent US-6027324-A) and commonly used in the 3D printing of three-dimensional resin models, is now being employed for the creation of 3D scaffolds with biomaterials. Inkjet printing and micro-extrusion are the most widely used printing techniques in bioprinting because, unlike stereolithography or fused deposition modeling, they do not use high temperatures or other processes that could deteriorate the viability of the cells contained in the bioinks [39].

### 3.1. Inkjet Bioprinting

**Figure 3:**
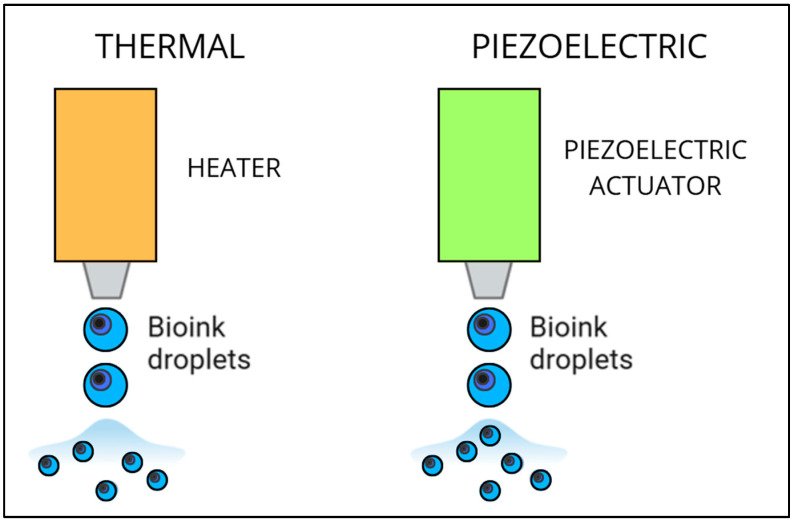
Schematic representation of inkjet bioprinting techniques: thermal process and piezoelectric process [31,78] (image created utilizing Canva^®^).

Inkjet bioprinting involves the ejection of the cell suspension by thermal or piezoelectric processes. In the thermal process, heat is used (temperature ranges from 100 to 300 °C) to create high pressure in the extruder, causing a droplet of bioink to be ejected. According to some studies, heat at these temperatures would not appear to compromise or, worse yet, degrade the bioink. While the piezoelectric process involves the use of acoustic waves to expel the bioink, it demonstrates the impossibility of using viscous inks. For the latter, micro-extrusion has proven to be better (Figure 3) [31,78].

### 3.2. Laser-Assisted Bioprinting

**Figure 4:**
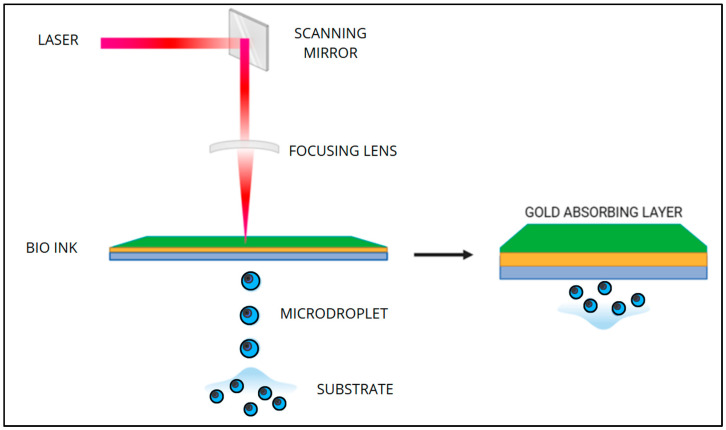
Schematic representation of laser-assisted bioprinting [79,80,81] (image created utilizing Canva^®^).

Laser-assisted bioprinting uses a beam of UV or visible light that can evaporate bioink placed on a ribbon. Once hit, the bioink evaporates and forms droplets that are deposited. UV light is also useful for activating molecules and photo-initiators, which can polymerize monomers and create reactive agents. A limitation of this technology is the cytotoxic effect of UV on the cells contained in the bioinks (Figure 4) [79,80,81].

### 3.3. Micro-Extrusion Bioprinting

**Figure 5:**
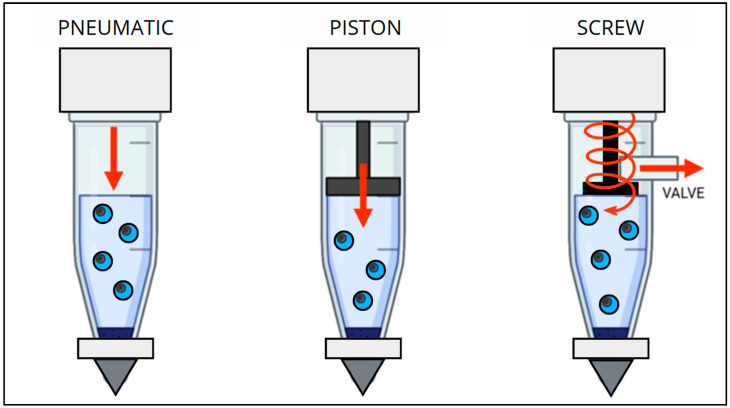
Schematic representation of micro-extrusion bioprinting [84,85] (image created utilizing Canva^®^).

Micro-extrusion bioprinting is the most popular method. It is based on pressure technology, where the bioink filament is ejected by means of a nozzle onto the substrate through pneumatic or mechanical pressure. This system is configured to a deposit continuous layer of bioinks to form the 3D construct. Once it is extruded, the material is solidified in many ways, such as chemical induction, heat, or photoinduced crosslinking. One of the problems with this printing methodology is the limited resolution [82,83]. This kind of technique employs mechanical forces to extrude the ink, and these forces can be classified into three categories: pneumatic, piston and screw-driven. The first one uses pressurized air to drive the ink, while the second and the last ones use a piston or a screw to extrude the bioink, providing a larger mechanical force and the best control during the printing (Figure 5) [84,85].

## 4. Applications in the Field of Maxillofacial Surgery

Three-dimensional bioprinting is emerging as a technology that can revolutionize regenerative medicine and reconstructive surgery. In fact, it can contribute significantly to the regeneration of craniofacial tissues and organs. In the field of maxillofacial surgery, there are several procedures characterized by major demolitions, particularly surgeries designed to remove neoplasms. Currently, to reconstruct an anatomical area surgically demolished due to neoplasm in place, there are several surgical techniques, including flaps, such as fibula flap, scapula flap, and many others [86].

The possibility of being able to construct portions or entire organs and tissues from living cells taken from the patient to reconstruct the demolished anatomical area would mean revolutionizing reconstructive surgery. There are several studies documenting the possible applications of this technology in the reconstruction of anatomical structures, such as bone defects, the regeneration of the temporomandibular joint disk, and fibrocartilage tissue. However, difficulties remain in the regeneration of tissues such as vascular tissue, particularly for small-caliber vessels such as capillaries [87,88,89,90].

To summarize the possible and promising applications in the maxillofacial field, 3D bioprinting proves to be a potentially useful tool for bone reconstruction, soft tissue regeneration, the study of neoplastic pathologies through tissue engineering methods, pharmaceutical experimentation, the reconstruction of the dentin–pulp complex, and skin and cartilage development. These are just a few examples of applications of this technology in tissue reconstruction of the head and neck region. The use of 3D printing in the future will surely revolutionize not only regenerative medicine and in vitro research studies of diseases and drugs but also surgery, particularly reconstructive surgery.

### 4.1. Dentin–Pulp Complex

Loss or damage to dental elements can pose a risk to oral health, often if these events are associated with pathological and inflammatory processes and phenomena [91]. Regarding the dentin–pulp complex, there are several studies, such as the one conducted by Han et al. (2019) [35], according to whose research bioprinting by the differentiation of dental-pulp stem cells can reproduce specific tissues capable of reconstructing dental elements. The study conducted by Qian et al. [92] through a Digital Light Processing (DLP) technique used GelMA (Gelatin Meth-acryloyl)-based bioink along with human dental-pulp stem cells for dental-pulp regeneration. Another recent study conducted by Liu et al. (2023) [93] through extrusion-based bioprinting increased the amount of keratinized gingiva in vivo using a bioink based on decellularized extracellular matrix, gelatin, and alginate, while an example of using synthetic polymers is the study conducted by Cho et al. (2016) [94], in which PCL (poly-ε-caprolactone) along with periodontal ligament stem cells was used to make cementum layers on the dentin surface. While a further study specific to the dentin–pulp complex, conducted by Nejad et al. (2021) [95], used PCL together with human dental-pulp stem cells to regenerate dentin and dental pulp in vitro via the extrusion-based bioprinting technique.

### 4.2. Skin

The skin is the most exposed organ in our body and is the most sensitive area in terms of aesthetic results. Tissue engineering in this field has already achieved important successes, such as the fabrication of artificial dermal grafts that can improve and facilitate healing, such as the fabrication of dermal substitutes consisting of human keratinocytes embedded with gelatinous substances. In addition, bioprinting is proving to be efficient in the reconstruction of multi-layered tissues in order to reproduce the exact histological anatomy of the skin by exploiting cells of different natures taken from the patient. Bioprinting would allow for the fabrication of realistic, customized skin substitutes, improving healing [96,97]. There are several studies that have made prints of skin constructs, such as the study conducted by Cubo et al. (2016) [98] that demonstrated it is possible to achieve bioprinted human skin differentiation through the use of human fibroblasts and keratinocytes within a fibrin-based hydrogel by extrusion technique. A recent study, conducted by Hafa et al.(2024) [99], applied the technique of light-based bioprinting (LiBB), demonstrating how LiBB is able to make skin constructs with precise tissue architectural features through the use of human fibroblasts and keratinocytes immersed in the GelMA (Gelatin Meth-acryloyl) hydrogel. In contrast, another study, conducted by Lee et al. (2024) [100], applied an additional printing technique called droplet-based bioprinting, making constructs that accurately mimic the morphology of native skin and promote healing mechanisms by adopting the use of primary human neonatal dermal fibroblasts immersed in a hydrogel consisting of type I collagen.

### 4.3. Bone Tissue

Bones are particularly present in the head and neck region, and given their complexity, bioprinting is a useful tool in the reconstruction of bone defects. Usually, when the defect exceeds 6 cm, the bone is hardly able to be repaired. For example, in demolition surgeries of the mandible, when it is the site of neoplasm, fibula flaps are used for reconstruction. This flap does not result in long-term gait disturbance, but lengthy rehabilitation is required before the patient can return to ambulation. Bioprinting may be able to construct the bone defect to be implanted in the patient from a cartilage model that, during development, acquires vascularization and replacement from the bone matrix [101,102]. The creation of bone tissues or their regeneration is not simple; indeed, the fabrication of these constructs turns out to be one of the most complex ever [103]. The generation of composite tissues, such as bone, remains significantly more complex, despite the fact that bioprinting of the skin has achieved significant milestones, such as the fabrication of multi-layered dermal and epidermal structures. The integration of mineralized matrices, vascular networks, and biomechanical properties that resemble native tissue is necessary for the formation of bone tissue. In contrast, skin bioprinting is a more accessible application of the technology, as it is based on simplified structural and cellular compositions. The integration of vascularization within the printed construct and the simultaneous maturation of multiple cell types are ongoing challenges in composite tissues [104,105]. Several studies have used biomaterials based on alginate, GelMA (Gelatin Meth-acryloyl), PEG (poly-ethylene glycol), and PCL (poly-ε-caprolactone) for the fabrication of bone constructs with the aim of being able to apply them in different areas, both in cranio-maxillofacial regeneration, but also in the treatment of metastatic bone lesions due to carcinomas such as breast cancer, for the regeneration of endochondral bone, and even in the repair of bone fractures, such as those of radial bone [106,107,108].

### 4.4. Intra-Oral Mucosa Tissue

An additional site of lesions, particularly neoplastic, is the intra-oral mucosa. In fact, mucosal defects can be numerous and diverse, oncologic, traumatic, infectious, and radiotherapeutic. Mucosal flaps or grafts are also currently used for the reconstruction of mucosal defects. However, it is not always possible to perform a reconstruction that can guarantee optimal functional and aesthetic performance. Mucosal bioprinting turns out to be particularly complex, given the anatomical complexity. Several studies have tried to take primary keratinocytes and fibroblasts via autologous biopsies and seed them into scaffolds for mucosal reconstruction. Some of these studies, exploiting dermal endothelial cells, have even succeeded in reconstructing pre-vascularized mucosa equipped with a capillary network [109,110,111]. For the repair and regeneration of injured oral mucosa, bioprinting can be used to make medical scaffolds for the purpose of delivering drugs to the site and environment of the injury, as was demonstrated in vitro by the study conducted by Koopaie et al. (2022) [112].

### 4.5. Cartilage Tissue

Finally, one of the tissues most studied by tissue engineering is cartilage tissue. In the head and neck district, cartilage tissue reconstruction can be useful in the rebuilding of auricular cartilage and nasal cartilage. There are studies that have used chondrocytes and stromal cells taken from adipose tissue immersed in poly-caprolactone and polyethylene glycol hydrogels for the reconstruction of auricular cartilage [113]. Although cartilage tissue repair is a significant clinical problem, 3D bioprinting represents an emerging technology for making autologous grafts for hyaline cartilage regeneration [114].

## 5. Limitations

Although research on 3D bioprinting is showing positive signs, there are important limitations that hinder its applicability in clinical reality, and one of them, the most important, is the realization of dense vascularized networks of small caliber. When exceeding 100–200 μm, bio-printed tissues require a vascular network, since oxygen diffusion occurs only within this limit. The fabrication of small vessels such as capillaries needs a resolution of 3 μm. Still, this goal has not yet been achieved since the highest resolution that can be achieved by laser bioprinting is 20 μm. Failure to vascularize tissues well results in necrosis or incomplete tissue development. Another key aspect to be taken into account is the properties of the endothelium, which has to fulfill the barrier function for the removal of waste products, coagulation, and inflammatory functions essential for proper tissue development and survival [115,116,117].

One challenge is the type of material used for bioprinting. One of the problems with commonly used biomaterials is that they can generate unwanted cellular interactions and cause problems in stem cell differentiation. In addition, some biomaterials are not suitable for conventional printing methods [118,119]. Another aspect to consider for this type of printing is the time required for the fabrication of these tissues. In fact, time is an important element, especially when considering patients with neoplastic diseases who need demolition surgery to remove the neoplasm and reconstructive to confer functionality of the demolished area [120]. The cost of this technology must also be considered. The recent emergence of bioprinting and the creation of innovative biomaterials and bioinks implies a major price increase to obtain this technology and to be able to spread it to more centers. In fact, the diffusion of bioprinting may take time [121,122]. Direct printing can lead to cell impairment, but the use of hydrogels within bioinks leads to an improvement in cell viability. In fact, there are several strategies to improve the printability of bioinks, shape fidelity, and cellular vitality and function. For example, mixing with biopolymers with cell-binding domains can improve cell binding and viability, while the presence of vascularized constructs can ensure better diffusion characteristics [123,124,125].

An important challenge in the field of bioprinting is the fabrication of bone tissues. The inherent cellular and mechanical complexities of bone and composite tissues present significant challenges in the bioprinting process. To replicate the hierarchical structure of bone, it is essential to employ a variety of cell types, such as osteoblasts, chondrocytes, and endothelial cells. In addition, not all biomaterials have strong mechanical properties, and this is a major obstacle. Although sophisticated bioreactors have been created to facilitate tissue maturation by creating controlled environments for nutrient delivery and mechanical stimulation, their potential applications are still limited to experimental settings. Further research is essential to incorporate bioreactors into scalable bioprinting systems for clinical applications.

Bioprinting’s current applicability is limited to experimental, preclinical, and in vitro studies, despite its significant potential in regenerative medicine. The complexity of this technology is emphasized by the challenges associated with vascularity, material properties, and, not least, regulatory barriers. As a result, bioprinting should not be considered a simple or cost-effective and clinically feasible solution at this time. Rather, it is a field that is in an evolving stage and requires significant research, increased investment, and interdisciplinary, multicenter collaboration to overcome the current obstacles.

## 6. Conclusions

Three-dimensional bioprinting is an innovative frontier in regenerative medicine and reconstructive surgery, offering new opportunities for the regeneration and repair of complex tissues damaged and degenerated by pathological processes and aging. This technology would allow for the creation of customized three-dimensional structures while improving aesthetic and functional outcomes.

Although bioprinting is generating great success in medicine and tissue engineering, limitations, such as realizing a vascular network of printed tissues, persist. Future research should focus on developing innovative techniques for functional vascular networks. The most promising strategies would seem to be using angiogenic factors and advanced bioreactors capable of creating vascularized tissue.

Three-dimensional bioprinting remains an innovative frontier in regenerative medicine, with potential for maxillofacial surgery, as of 2025. Over the years, there has been an increase in knowledge, the adoption of hybrid printing techniques, and the integration of nanomaterials in order to improve the stability and integrity of printed structures. Nevertheless, substantial limitations continue to exist. These hurdles must be overcome to transform bioprinting from an experimental technology to a viable clinical tool. Future research must focus on this goal. The progress made to date is promising; however, it is imperative to maintain interdisciplinary efforts in order to fully realize the potential of bioprinting in maxillofacial reconstruction.

## Figures and Tables

**Table 1:** More applications of 3D printing in cranio-maxillofacial surgery [14].

Applications of 3D Printing	Functions
Surgical guide	They are useful instruments to guide the surgeon during the execution of the osteotomy, such as in orthognathic surgery.
3D models	The creation of 3D models of complex anatomical parts can be useful for the surgeon to pre-operatively plan the operation and better understand the type of approach to be taken, and can serve an educational function for young surgeons and students.
Patient-specific implants	These types of 3D models are customized and implantable tools. They make it possible to conduct a repair of the anatomical area involved in trauma or for bone reconstructions following a demolition operation, as is the case for some cancer patients.
Splints	These customized models make it possible to guide movements and stabilize the patient’s occlusion during orthognathic surgery.
Molds	Molds are useful tools for making cranioplasties. It is possible to make molds with non-implantable material in order to model implantable materials such as PMMA (polymethylmethacrylate) in neurosurgery.

**Table 2:** The characteristics of the main natural polymers used in bioprinting.

Natural Polymer	Main Features
Alginate [43,44,45]	- Commonly used; - Easy printability; - Good water absorption; - Low cost; - Compatibility with many tissues.
Agarose [46,47,48]	- Limited cellular support function; - Controllable with temperature; - “sacrifice biomaterial”.
Collagen [49,50,51]	- Influences cell adhesion; - Controllable with pH and temperature; - Poor mechanical strength.
Gelatin [52,53,54]	- Low immunogenicity; - Biodegradable; - Good biocompatibility; - Need for additional materials to increase stability.
Chitosan [55,56,57,58]	- Antibacterial function; - Biodegradable; - Non-toxic properties; - Not excessively expensive; - Low mechanical strength.
Hyaluronic acid [59,60,61]	- Good water absorption; - Good cellular adaptation; - Poor mechanical strength.
Fibrin [62,63,64,65]	- Good cellular adaptation; - Poor mechanical strength; - Rapid degradation.
Decellularized extracellular matrix [66,67,68,69,70]	- Good cellular support; - Poor mechanical strength; - Applicable in various tissues.

**Table 3:** The characteristics of the main synthetic polymers used in bioprinting.

Synthetic Polymers	Main Features
Poly-ethylene glycol (PEG) and poly-ethylene oxide (PEO) [72,73,74]	- Excellent water absorption; - Poor immunogenicity; - Good biocompatibility; - High mechanical strength; - High permeability; - Poor cell adhesion.
Poly-ε-caprolactone (PCL) [75,76,77]	- Durable; - Biodegradable; - High mechanical strength; - Often used for bone tissue.

## Data Availability

The data are available upon request.
